# The inhibitory avoidance discrimination task to investigate accuracy of memory

**DOI:** 10.3389/fnbeh.2015.00060

**Published:** 2015-03-12

**Authors:** Erika Atucha, Benno Roozendaal

**Affiliations:** ^1^Department of Cognitive Neuroscience, Radboud University Medical CentreNijmegen, Netherlands; ^2^Donders Institute for Brain, Cognition and Behaviour, Radboud University NijmegenNijmegen, Netherlands

**Keywords:** accuracy, context memory, discrimination, episodic-like memory, inhibitory avoidance, norepinephrine, yohimbine

## Abstract

The present study was aimed at developing a new inhibitory avoidance task, based on training and/or testing rats in multiple contexts, to investigate accuracy of memory. In the first experiment, male Sprague-Dawley rats were given footshock in an inhibitory avoidance apparatus and, 48 h later, retention latencies of each rat were assessed in the training apparatus (Shock box) as well as in a novel, contextually modified, apparatus. Retention latencies in the Shock box were significantly longer than those in the Novel box, indicating accurate memory of the training context. When the noradrenergic stimulant yohimbine (0.3 mg/kg, sc) was administered after the training, 48-h retention latencies in the Shock box, but not Novel box, were increased, indicating that the noradrenergic activation enhanced memory of the training experience without reducing memory accuracy. In the second experiment, rats were trained on an inhibitory avoidance discrimination task: They were first trained in an inhibitory avoidance apparatus without footshock (Non-Shock box), followed 1 min later by footshock training in a contextually modified apparatus (Shock box). Forty-eight-hour retention latencies in the Shock and Non-Shock boxes did not differ from each other but were both significantly longer than those in a Novel box, indicating that rats remembered the two training contexts but did not have episodic-like memory of the association of footshock with the correct training context. When the interval between the two training episodes was increased to 2 min, rats showed accurate memory of the association of footshock with the training context. Yohimbine administered after the training also enhanced rats' ability to remember in which training context they had received actual footshock. These findings indicate that the inhibitory avoidance discrimination task is a novel variant of the well-established inhibitory avoidance task suitable to investigate accuracy of memory.

## Introduction

Inhibitory avoidance is a commonly used behavioral task to investigate learning and memory processes in rodents (Gold, 1986; McGaugh and Roozendaal, 2009). During inhibitory avoidance training, rats typically receive a single aversive footshock after stepping from a lighted compartment into a darkened compartment in a straight alley. Retention of the training is tested usually 24 or 48 h later by measuring rats' latency to enter the former shock compartment when they are placed in the lighted compartment. Longer retention test latencies are interpreted as indicating better memory. Even though inhibitory avoidance training consists of a single trial only, the brain processes underlying task acquisition are complex. Rats must encode different pieces of information in order to acquire a correct association between a particular location within the apparatus and the aversive stimulus of footshock (Liang, 2001; Malin and McGaugh, 2006; Roozendaal and McGaugh, 2011; Fornari et al., 2012). During the past decades we have learned much regarding the involvement of specific brain regions and neurochemical processes in different aspects of inhibitory avoidance memory (Izquierdo et al., 2006; Roozendaal and McGaugh, 2011). However, with these inhibitory avoidance training and test procedures it is difficult, if not impossible, to make any inference with respect to the accuracy or specificity of what has been learned. Better memory, i.e., longer retention test latencies, on the inhibitory avoidance task as induced, for example, by posttraining drug manipulation could indicate that the rat developed a more accurate or detailed representation of the training experience. On the other hand, it is also possible that the memory enhancement is associated with reduced accuracy. Particularly in the field of stress research on cognitive processes there is currently much interest in unraveling how stress and emotional arousal affect both the strength and accuracy of memory processing (Heuer and Reisberg, 1990; Payne et al., 2002; Segal et al., 2012; Hoscheidt et al., 2014; Leal et al., 2014). Moreover, recent developments in *in vivo* neuroimaging, optogenetics, and electrophysiological techniques indicate the necessity of having refined behavioral tasks that allow investigating the neural substrates underlying specific aspects of information processing and memory (Rauch et al., 2006; Laxpati et al., 2014).

The present study was undertaken to establish and validate an inhibitory avoidance discrimination task to investigate accuracy of both contextual and episodic-like aspects of memory. In the first series of experiments the inhibitory avoidance training procedure was left unchanged, but retention latencies of each rat were assessed in the training apparatus in which they had received footshock as well as in another, contextually modified, inhibitory avoidance apparatus they had not seen during the training. This allows investigating whether the brief context exposure during training is sufficient for rats to develop an accurate memory of the training context. In the second series of experiments, rats were subsequently trained in two distinctly different inhibitory avoidance apparatuses with a short delay, but were given footshock in only one of these contexts. On the retention test, the rats were tested in these two training contexts as well as in a novel context. This training and test procedure allows investigating whether rats remember the two contexts they visited during the training as well as have specific episodic-like memory of the association of footshock with the correct training context. Our main objective was to develop a study protocol which is highly ambiguous and induces poor discrimination in control animals, such that it can be examined to what extent memory-enhancing drug treatment also increases the accuracy of memory. As norepinephrine, normally released by emotionally arousing training, is known to enhance the consolidation of inhibitory avoidance memory (Liang et al., 1986; Introini-Collison et al., 1991; Ferry et al., 1999; McIntyre et al., 2005), we investigated, as proof-of-principle, on both tasks how posttraining systemic administration of a memory-enhancing dose of the noradrenergic stimulant yohimbine might affect the accuracy and/or strength of the memory.

## Materials and methods

### Subjects

Adult male Sprague-Dawley rats (330–370 g at the time of behavioral experiments) from Charles River Breeding Laboratories (Kisslegg, Germany) were housed individually in a temperature-controlled (22°C) vivarium room and maintained on a 12-h/12-h light/dark cycle (lights on: 7:00–19:00 h) with *ad libitum* access to food and water. Rats were handled three times for 1 min each prior to training. Training and testing were performed during the light phase of the cycle, between 10:00–15:00 h. All experimental procedures were in compliance with the European Communities Council Directive of November 24, 1986 (86/609/EEC) and approved by the Institutional Animal Care and Use Committees of the University of Groningen and Radboud University Nijmegen, The Netherlands.

### Inhibitory avoidance discrimination task

#### Inhibitory avoidance apparatus and contextual modifications

**Figure 1:**
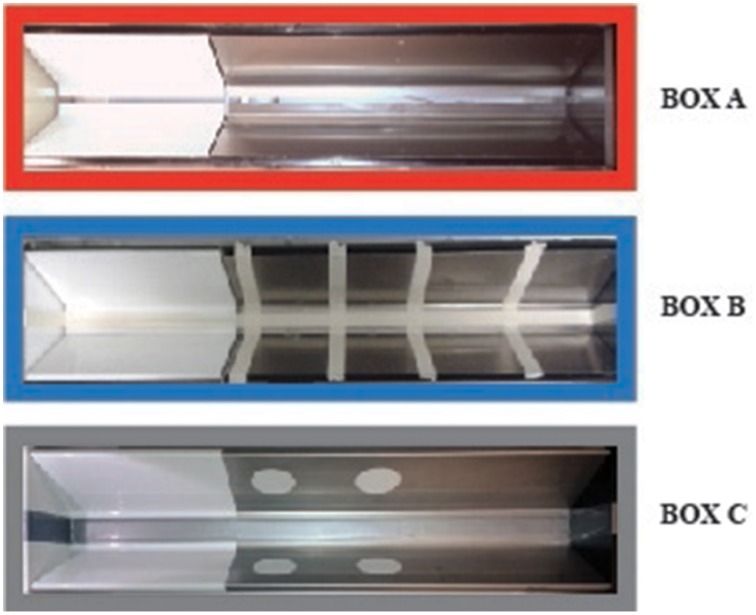
**Inhibitory avoidance apparatus and contextual modifications**. Box **(A)** (red frame) did not have any contextual modifications. Footshock was delivered in this apparatus only. Box (**B,C)** had some distinct contextual modifications and served as non-shock safe training and/or test contexts. Box **(B)** (blue frame) had four vertical white stripes taped on the wall of the dark compartment together with tape placed on the floor, closing the gap between the two plates along the entire length of the apparatus. Box **(C)** (gray frame) had two white circles taped on each wall of the dark compartment, and the gap between both plates was closed with tape. The colored frames refer to the diagrams in **Figures 2, 3**.

For all experiments, rats were trained and tested in one or more inhibitory avoidance apparatuses. The geometry and basic features of each apparatus were identical and consisted of a trough-shaped alley (91 cm long, 15 cm deep, 20 cm wide at the top, and 6.4 cm wide at the bottom) divided into two compartments, separated by a sliding door that opened by retracting into the floor (McGaugh et al., 1988). The starting compartment (31 cm) was made of opaque white plastic and was well lit; the dark (i.e., shock) compartment (60 cm) was made of two electrifiable metal plates and was not illuminated. As shown in Figure 1, one apparatus (Box A) did not have any contextual modifications. Footshock was always delivered in this apparatus only. Two other apparatuses (Boxes B,C) served as non-shock, safe training or test environments and had some distinct contextual modifications. Box B had four vertical white stripes (2 cm wide) taped on the wall of the dark compartment together with tape placed on the floor, closing the gap between the two plates along the entire length of the apparatus. Box C had two white circles (3.5 cm diameter) taped on each wall of the dark compartment, and the gap between both plates was closed with tape. All three inhibitory avoidance apparatuses were located next to one other within a sound- and light-attenuated room.

#### Procedures of the inhibitory avoidance task

In the first series of experiments, rats were trained in Box A, as with the classical inhibitory avoidance training procedure, and subsequently tested for retention, 48 h after training, in that same apparatus and a novel apparatus. The addition of a novel test environment during the retention test permits to determine whether the rats accurately remembered the context in which they had received footshock. Moreover, we examined in this experiment what the best procedure might be for assessing retention latencies of individual rats on both apparatuses. For training, the rats were placed into the starting compartment of Box A (Shock box), facing away from the door, and were allowed to freely explore the apparatus. After the rat stepped completely into the dark compartment, the sliding door was closed and a single inescapable footshock (0.50 mA for 1 s) was delivered. Rats were removed from the dark compartment 20 s later and returned to their home cages until retention testing 48 h later. For retention testing, the rats were placed, in a pseudo-random fashion, into the starting compartment of either Box A or a novel apparatus (Box B) and their latencies to enter the dark compartment with all four paws (maximum latency of 600 s) were measured. Shock was not administered on the retention test trial. Either immediately or 24 h after testing in the first apparatus (see Results), the same rats were placed into the starting compartment of the other apparatus and their latencies to enter the dark compartment were measured. Immediately after the training or testing of each animal, the apparatuses were wiped clean with a 10% ethanol solution.

#### Procedures of the inhibitory avoidance discrimination task

In the second series of experiments, rats were trained subsequently in two inhibitory avoidance apparatuses within a single training session, but footshock was delivered only in one of these two contexts. On the 48-h retention test, their latencies to enter the dark compartment of these two boxes were determined as well as those into that of a novel apparatus they had not seen before. This experimental design was aimed at investigating whether rats, on a retention test, would remember the two apparatuses they had visited during training and discriminate in which of these two contexts they had received actual footshock. Rats were initially placed into the starting compartment of either Box B or C (Non-Shock box) and could explore this apparatus for 20 s without any footshock being delivered. Afterwards, the rats were taken from that apparatus and, after a brief delay of either 1 or 2 min, placed into the starting compartment of Box A (Shock box). After entering the dark compartment of Box A, the sliding door was closed and a single inescapable footshock of 0.5 mA was delivered for 1 s. Rats were removed from the dark compartment 20 s after termination of footshock and returned to their home cages. The order of training on these two boxes was always the same. Rats were first trained in the Non-Shock box followed by the Shock box because they are less likely to explore a new environment shortly after shock delivery. During the 48-h retention test, the rats were tested in the previously seen Non-Shock and Shock boxes and, additionally, in a Novel box they had not seen before. The order of retention testing in these three contexts was randomized. On all three inhibitory avoidance apparatuses, the rat was placed into the starting compartment and their latency to enter the dark compartment with all four paws (maximum latency of 600 s) was measured. After the rat entered the dark compartment of the first test environment, it was immediately taken from that apparatus and without delay placed into the starting compartment of the second, and then third, box. Shock was not administered on the retention test trial. Immediately after the training or testing of each animal, the apparatuses were wiped clean with a 10% ethanol solution.

### Systemic drug administration

Some groups of rats received an immediate posttraining injection of a memory-enhancing dose of the noradrenergic stimulant yohimbine (0.3 mg/kg; Sigma-Aldrich), a selective α_2_-adrenoceptor antagonist. Yohimbine was dissolved in sterile 0.9% saline and administered subcutaneously in a volume of 2 ml/kg immediately after the training session. Control animals received a saline injection only. The drug dose was selected on the basis of previous findings (Roozendaal et al., 2006). Drug solutions were freshly prepared before each experiment.

### Statistics

Data are expressed as the mean ± SEM. Retention test latencies were analyzed with one-, two- or three-way ANOVAs with latencies of individual animals in the different test environments (Shock, Non-Shock, and Novel boxes) as repeated measure. *Post-hoc* comparisons used unpaired and paired *t*-tests to determine the source of the detected significances, when appropriate. Training and retention latencies of each rat were compared with paired *t*-tests. For all comparisons, a probability level of <0.05 was accepted as statistical significance. The number of animals per group is indicated in the figure legends.

## Results

### Testing accuracy of inhibitory avoidance memory

**Figure 2:**
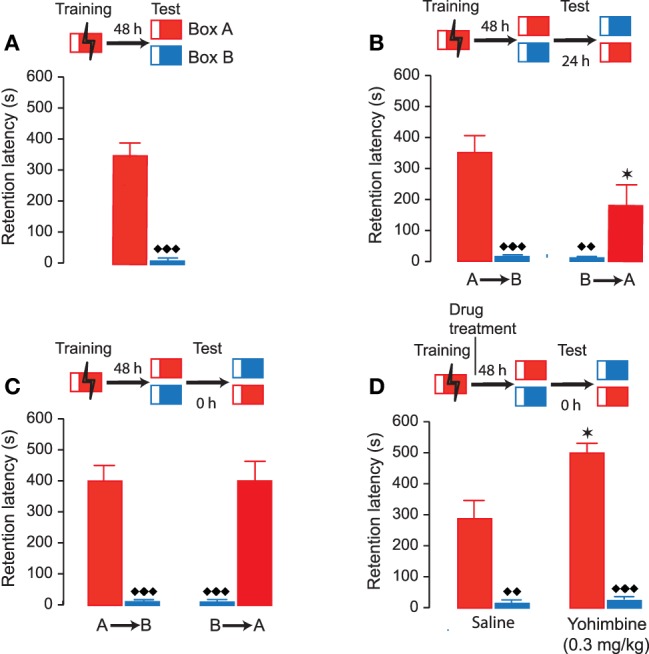
**Accuracy of inhibitory avoidance memory**. Inhibitory avoidance retention latencies (mean ± SEM) in seconds. The different test procedures are shown as schematic for each of the experiments. **(A)** Rats were given footshock in Box A (red) and 48 h later half of the rats were tested for retention in that same apparatus and the other half in a novel apparatus (Box B, blue). Retention latencies in Box A were significantly longer than retention latencies in Box B. ♦♦♦, *P* < 0.0001 vs. Box A. *N* = 8–9 rats/group. **(B)** Twenty-four hours later the same rats were tested in the other apparatus. Retention latencies in Box A were now significantly shorter than during the first retention test. A→B, rats were first tested in Box A and 24 h later in Box B; B→A, rats were first tested in Box B and 24 h later in Box A. ★, *P* < 0.05, vs. Box A during first retention test. ♦♦, *P* < 0.01; ♦♦♦, *P* < 0.0001 vs. Box A. **(C)** Rats were trained in Box A and 48 h later half of the rats were tested for retention in the same apparatus and the other half in a novel apparatus (Box B). They were immediately afterwards tested in the other apparatus. Retention latencies were now independent of the order of testing. A→B, rats were first tested in Box A and then in Box B; B→A, rats were first tested in Box B and then in Box A. ♦♦♦, *P* < 0.0001 vs. Box A. *N* = 9 rats/group. **(D)** Yohimbine (0.3 mg/kg, sc) administered immediately after inhibitory avoidance training enhanced 48-h retention latencies in Box A without affecting retention latencies in Box B. ★, *P* < 0.05 vs. the saline group. ♦♦, *P* < 0.01; ♦♦♦, *P* < 0.0001 vs. Box A. *N* = 8–9 rats/group.

The first experiment investigated whether rats after inhibitory avoidance training develop accurate memory of the context in which they received footshock. Therefore, rats were trained on the one-trial inhibitory avoidance task (Box A). Initial latencies to enter the dark compartment during training, before footshock, were 13.9 ± 2.1 s (mean ± SEM). Forty-eight hours later, half of the rats were tested in the same apparatus (Box A) and the other half in a distinctly different novel context (Box B). As shown in Figure 2A, retention latencies of rats tested in the Shock box (Box A) were significantly longer (350.4 ± 49.4 s) than those of rats that were tested in the novel context (Box B) (10.6 ± 1.7 s; *P* < 0.0001). Moreover, retention latencies of rats tested in Box A were significantly longer than their entrance latencies in that apparatus during the training trial (paired *t*-test: *t*_7_ = 6.84; *P* = 0.0005), whereas retention latencies of rats tested in Box B did not differ from their prior training latencies in Box A (paired *t*-test: *t*_8_ = 0.59; *P* = 0.57). Thus, these findings indicate that rats showed accurate memory of the context in which they received footshock.

Twenty-four hours after the first retention test, the same animals were tested in the other inhibitory avoidance apparatus. Thus, rats that had been tested in Box A were now tested in Box B, and rats that had been tested in Box B were now tested in Box A. Again, we found that rats had significantly longer retention latencies when tested in Box A as compared to Box B (*P* < 0.01; Figure 2B). However, retention latencies in Box A were now significantly shorter than those during the first retention session (*P* < 0.05). In a next experiment, another group of animals was trained in Box A and 48 h later tested for retention in both inhibitory avoidance apparatuses, but now without any delay. Rats were tested, in a randomized fashion, in either Box A or B and immediately afterwards in the other context. As shown in Figure 2C, retention latencies in Box A were significantly longer than those in Box B (*P* < 0.0001). Most importantly, retention latencies in Box A and B were now independent of the order of testing (repeated-measures ANOVA: *F*_1,16_ = 0.0005; *P* = 0.98). These findings indicate that repeated testing of the same animals in both inhibitory avoidance apparatuses without a delay is an accurate method for assessing discrimination in individual rats. Therefore, for all further experiments retention latencies in the different test environments were determined without delay.

As we are particularly interested in investigating how posttraining drug treatment might affect accuracy of memory, we examined whether noradrenergic stimulation after inhibitory avoidance training would enhance memory of the training in a context-specific manner. Rats were trained on the inhibitory avoidance task (Box A) and given a posttraining subcutaneous injection of either saline or memory-enhancing dose of the noradrenergic stimulant yohimbine (0.3 mg/kg). Rats were tested 48 h later for retention in both Box A and B. The order of retention testing in these two contexts was randomized and without any delay. As shown in Figure 2D, yohimbine increased retention latencies in the Shock box (*P* < 0.05 vs. saline) without influencing retention latencies in the Novel box (*P* = 0.84 vs. saline). Moreover, repeated-measures ANOVA indicated that successive testing did not affect retention latencies (*F*_1, 15_ = 1.57; *P* = 0.23). Thus, these findings indicate that yohimbine enhanced memory of the training experience without inducing any generalization across contexts.

### Testing accuracy of inhibitory avoidance discrimination memory

**Figure 3:**
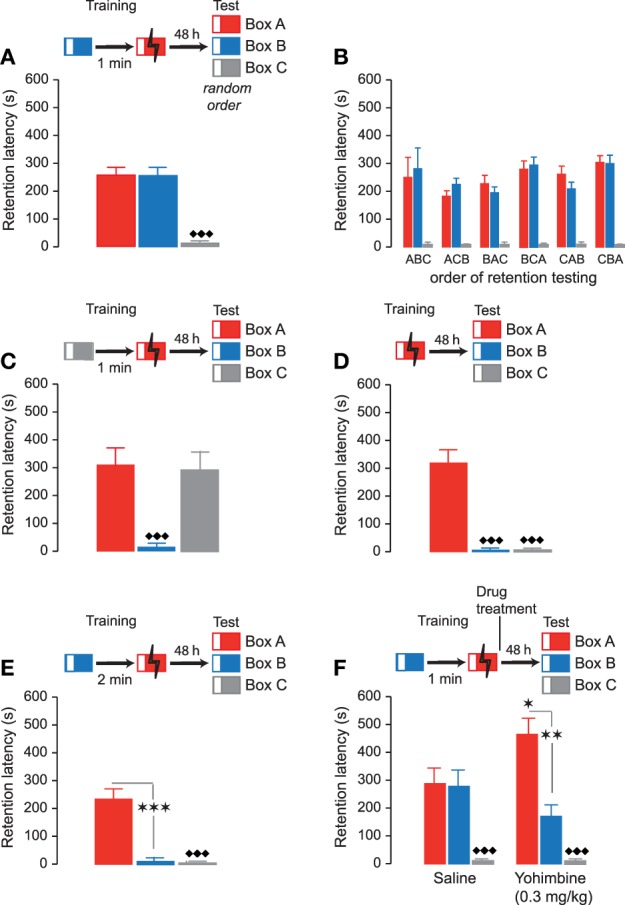
**Accuracy of inhibitory avoidance discrimination memory**. Inhibitory avoidance retention latencies (mean ± SEM) in seconds. The different training procedures are shown as schematic for each of the experiments. **(A)** Rats were trained in Box B (Non-Shock box, blue) without footshock followed 1 min later by footshock training in Box A (Shock box, red). On the 48-h retention test rats were sequentially tested in all three inhibitory avoidance apparatuses in a random order and without delay. Retention latencies in the previously visited Box A and Box B did not differ from each other but were both significantly longer than those in Box C (Novel box, gray). ♦♦♦, *P* < 0.0001 vs. Box A and Box B. *N* = 30 rats. **(B)** The order of retention testing in the three boxes (ABC, ACB, BAC, BCA, CAB, CBA) did not significantly influence retention latencies. **(C)** On the training trial rats were first placed in Box C (Non-Shock box) followed 1 min later by footshock training in Box A (Shock box). Forty-eight-hour retention latencies in the two previously visited boxes (Box A and Box C) did not differ from each other but were both significantly longer than those in Box B (Novel box). ♦♦♦, *P* < 0.0001 vs. Box A and Box C. *N* = 13 rats. **(D)** Rats were trained in Box A (Shock box). Forty-eight-hour retention latencies in Box A were significantly longer than those in Box B and C. ♦♦♦, *P* < 0.0001 vs. Box A. *N* = 20 rats. **(E)** When the interval between training in Box B (Non-Shock box) and Box A (Shock box) was 2 min, 48-h retention latencies in Box A were significantly longer than those in Box B and Box C (Novel box). ★★★, *P* < 0.0001 vs. Box A. ♦♦♦, *P* < 0.0001 vs. Box A. *N* = 15 rats. **(F)** Yohimbine (0.3 mg/kg, sc) administered immediately posttraining enhanced 48-h retention latencies for the Shock box (Box A). Retention latencies in Box A (Shock box) were significantly longer than those in Box B (Non-Shock box) and Box C (Novel box). ★, *P* < 0.05 vs. the saline group. ★★, *P* < 0.01 vs. Box A. ♦♦♦, *P* < 0.0001 vs. Box A. *N* = 12 rats/group.

In the second series of experiments, rats were subsequently trained in two inhibitory avoidance apparatuses within a single training session, but footshock was delivered only in one of these two contexts. During the training session, rats were first trained in Box B (Non-Shock box) and 1 min later in Box A (Shock box). On the 48-h retention test, rats were tested, in a randomized order and without delay, in the Shock and Non-Shock boxes as well as in a Novel box (Box C) they had not seen during the training. Repeated-measures ANOVA for retention test latencies in these three boxes indicated a significant context effect (*F*_2, 48_ = 52.53, *P* < 0.0001). As shown in Figure 3A, retention latencies in the Shock box (Box A: 255.8 ± 29.9 s) and Non-Shock box (Box B: 255.1 ± 30.4 s) did not differ from each other (paired *t*-test: *t*_29_ = 0.63; *P* = 0.53), but were both significantly longer than those in the novel context (Box C: 13.4 ± 1.5 s; paired *t*-tests: Shock box vs. Novel box: *t*_29_ = 7.99; *P* < 0.0001; Non-Shock box vs. Novel box: *t*_29_ = 7.84; *P* < 0.0001). Moreover, as shown in Figure 3B, retention latencies in the three test contexts were independent of the order of testing (ABC, ACB, BAC, BCA, CAB, CBA: *F*_5, 24_ = 0.26, *P* = 0.93). Thus, these findings indicate that with this training procedure rats accurately remembered the two contexts they had visited during the training but lack specific memory of in which training context they had received actual footshock.

To determine whether the difference between retention latencies in Box B and Box C, which were both safe contexts, might have been caused because the contextual modifications made them not equally distinct from Box A, two additional experiments were performed. In the first experiment, Box C now served as Non-Shock context and Box B as Novel context. Rats were trained in Box C (Non-Shock box) followed 1 min later by footshock delivery in Box A (Shock box). Forty-eight hours later, rats were tested in all three contexts in a randomized manner and without delay. As shown in Figure 3C, repeated-measures ANOVA for retention latencies in the three apparatuses revealed a significant context effect (*F*_2, 14_ = 37.83, *P* < 0.0001). Retention latencies in the Shock box (Box A) and Non-Shock box (Box C) did not differ from each other (paired *t*-test: *t*_12_ = 0.77; *P* = 0.45) but were both significantly longer than those in the Novel box (Box B) (paired *t*-tests: Shock box vs. Novel box: *t*_12_ = 5.95; *P* < 0.0001; Non-Shock box vs. Novel box: *t*_12_ = 6.75; *P* < 0.0001). Thus, these findings indicate that rats had similar retention latencies in the two apparatuses (Shock box and Non-Shock box) they had visited on the training trial, irrespective of whether the Non-Shock box was Box B or C. To further examine whether Box B and C were both sufficiently distinct from Box A, rats were trained in Box A (Shock box) only and tested 48 h later in all three contexts in a randomized manner. Repeated-measures ANOVA for retention latencies indicated a significant context effect (*F*_2, 28_ = 54.05, *P* < 0.0001). As shown in Figure 3D, retention latencies in Box A (322.1 ± 41.6 s) were significantly longer than those in Box B (9.3 ± 0.9 s; paired *t*-test: *t*_19_ = 7.15; *P* < 0.0001) and Box C (8.9 ± 1.0 s; paired *t*-test: *t*_19_ = 7.14; *P* < 0.0001). Moreover, retention latencies in Box B and C did not differ from each other (paired *t*-test: *t*_19_ = 0.84; *P* = 0.41). These findings indicating that rats readily discriminate the different test contexts, thus, strongly suggest that the similar long retention latencies in the Shock and Non-Shock boxes were caused because rats were unable to remember in which of the two training contexts they had received actual footshock.

To determine whether the difficulty of rats to associate the footshock experience with the correct training context might be due to the short interval between the two training episodes, causing a temporal overlap of the two memory traces, in the next experiment rats were trained in the Non-Shock (Box B) and Shock (Box A) boxes with an increased interval of 2 min. As shown in Figure 3E, repeated-measures ANOVA for 48-h retention latencies indicated a significant context effect (*F*_2,18_ = 46.75, *P* < 0.0001). Retention latencies in the Shock box (Box A) were now significantly longer than those in the Non-Shock box (Box B) (paired *t*-test: *t*_9_ = 5.89; *P* < 0.0001), indicating discrimination, whereas retention latencies for the Non-Shock and Novel boxes did not differ (paired *t*-test: *t*_9_ = 0.50; *P* = 0.63). Thus, the longer interval between the two context exposures at training made it easier for the rats to associate the footshock experience with the correct training context.

Finally, rats were trained on the inhibitory avoidance discrimination task, with a 1-min interval between both training episodes, and given an immediate posttraining subcutaneous injection of saline or memory-enhancing dose of yohimbine (0.3 mg/kg). Forty-eight hours later, retention was tested by successively testing rats in the three contexts (Box A, B and C), in a randomized fashion. Two-way repeated-measures ANOVA indicated a significant yohimbine effect (*F*_1,66_ = 3.12, *P* = 0.03) as well as a significant interaction between yohimbine treatment and test context (*F*_2,66_ = 7.08, *P* = 0.001). As shown in Figure 3F, saline-treated rats had similar retention latencies in the Shock and Non-Shock boxes (paired *t*-test: *t*_11_ = 0.21; *P* = 0.79), indicating lack of discrimination. Yohimbine treatment significantly increased retention latencies in the Shock box (*P* < 0.05 vs. saline). Moreover, and importantly, retention latencies in the Shock box (Box A) were significantly longer than those in the Non-Shock box (Box B) (paired *t*-test: *t*_11_ = 4.99; *P* < 0.01), indicating discrimination. Yohimbine treatment did not alter retention latencies in the Novel box (Box C) (*P* = 0.81 vs. saline). These findings thus indicate that the posttraining yohimbine administration enhanced rats' specific memory of the association of footshock with the correct training context. Again, we found no evidence that the yohimbine induced generalization across contexts.

## Discussion

The present series of experiments was aimed at establishing and validating an inhibitory avoidance discrimination task which allows investigating and manipulating accuracy of memory. Inhibitory avoidance is a commonly used behavioral task to investigate learning and memory processes in rodents. One of the great assets of this task is that a single footshock stimulation is sufficient to create robust long-term memory, making this task highly suitable to investigate drug effects on memory (Roozendaal and McGaugh, 2011). However, because retention of inhibitory avoidance training is usually tested only in the training context, it is difficult to define exactly what an animal has learned and whether a drug manipulation affected the accuracy of memory. The inhibitory avoidance discrimination task, which is based on training and testing rats in multiple contexts, does allow such more specific conclusions.

The first set of experiments was aimed at investigating whether the short context exposure during inhibitory avoidance training (typically less than 1 min) is sufficient to induce accurate memory of the training context. Therefore, rats were trained on the classical inhibitory avoidance task and retention was tested 48 h later in either the training apparatus or a novel, contextually modified, apparatus. This procedure of retention testing in the training context as well as a novel, safe context is sometimes used in contextual fear conditioning experiments (Wiltgen and Silva, 2007; Wang et al., 2009). However, a training session in contextual fear conditioning experiments typically involves multiple footshocks and lasts longer than with inhibitory avoidance. Moreover, the training and novel apparatuses in our experiments were very similar and differed only from each other by some tape placed on the walls and floor. Our finding indicating that rats had long retention latencies in the training context whereas they readily entered the dark compartment of the novel apparatus shows that the brief context exposure during training is sufficient to create robust and accurate memory of the training context. Next, we investigated whether it is possible to test the same rat in the other apparatus as well without altering retention latencies due to the repeated testing. When animals were tested with a 24-h delay in the other apparatus, retention latencies in the Shock box were significantly shorter in comparison to retention latencies in that same apparatus on the previous day. Such shorter retention latencies in Box A 24 h after rats had first been tested in a novel context (Box B) could be caused by different mnemonic processes such as extinction learning, safety learning or reconsolidation, that might all require memory consolidation (Cammarota et al., 2004; Alberini, 2011). However, when rats were tested for retention in both contexts without a delay, retention latencies of rats that were tested in the Shock box during the first or second test session were nearly identical. Thus, these findings indicate that repeated retention testing in both boxes without a delay is a suitable method for assessing accuracy of contextual memory in individual animals. To determine, as proof-of-principle experiment, whether it is possible to assess the effect of a posttraining pharmacological manipulation on memory accuracy, rats were treated with yohimbine after inhibitory avoidance training. We selected yohimbine for this experiment because of extensive evidence indicating that posttraining noradrenergic activation enhances the consolidation of inhibitory avoidance memory (Liang et al., 1986; Introini-Collison et al., 1991; Ferry et al., 1999; McIntyre et al., 2005). Our finding that yohimbine enhanced retention latencies in the Shock box but did not affect retention latencies in the Novel box indicates that the memory enhancement induced by yohimbine is not associated with generalization across contexts and, thus, reduced accuracy.

In the second set of experiments rats were trained on the inhibitory avoidance discrimination task. They were exposed to two inhibitory avoidance apparatuses, with a 1-min delay, but footshock was delivered only in one of the training contexts. Retention was tested 48 h later in both apparatuses as well as in a novel apparatus they had not seen before. Our finding that retention latencies in both the Shock box and Non-Shock box were significantly longer than those in a Novel box indicates again that rats had good memory of the two contexts they visited during the training. However, the similar retention latencies in the Shock and Non-Shock boxes further indicates that the presentation of the two training contexts with such a short interval did not allow rats to create accurate memory of the association of footshock with the actual training context. Importantly, the inhibitory avoidance discrimination task incorporates the critical element of contextual discrimination as an episode for the assessment of episodic-like memory in rats. Episodic memory refers to memory for an event that holds spatio-temporal relations (Tulving, 1983). When the interval between training on the two inhibitory avoidance apparatuses was increased to 2 min, retention latencies in the Shock box were significantly longer than those in the Non-Shock box and Novel box. Thus, these findings indicate that increasing the interval between the two training episodes facilitates discrimination between both events. Our finding that successful context discrimination can occur after a single exposure to the contexts is rather remarkable. Discriminatory study protocols involving aversive shock often require multiple trials, yet a large degree of generalization across different contexts is found (Chess et al., 2009; Czerniawski and Guzowski, 2014). In one study (Kim et al., 2013), rats were trained for several days on an auditory-cue fear conditioning task in two different contexts. In one training context the conditioning stimulus (CS) was consistently paired with the unconditioned stimulus of footshock, whereas in the other training context the CS-only was presented. Only after three training days rats were able to discriminate, but still rather poorly, between the shock and safe context. In contrast to this latter study, in our study design the footshock was directly associated with a particular training context which could explain the much more rapid emergence of context discrimination. In addition, the ability to discriminate most likely depends, among other factors, on the duration of context exposure as well as the level of contextual modification (Gozález et al., 2003; McHugh and Tonegawa, 2007). To test whether posttraining drug manipulation can modulate accuracy of the association of footshock with the specific training context, yohimbine was administered immediately after training on the inhibitory avoidance discrimination task. In contrast to saline-treated control rats, rats administered yohimbine after the training had significantly longer retention latencies in the Shock box than in the Non-Shock box. These findings thus strongly suggest that posttraining noradrenergic activation also facilitates the consolidation of memory of episodic-like aspects of the training. As a result, the subsequently formed memory yields a greater degree of accuracy. In agreement with these findings, we recently reported that norepinephrine infused posttraining into the basolateral amygdala enhanced memory precision in an object-in-context recognition task, increasing rats' ability to discriminate in which training context they had seen a particular object (Barsegyan et al., 2014). Conversely, blockade of noradrenergic transmission in the basolateral amygdala with posttraining infusions of propranolol impaired memory on this task, indicating that endogenous noradrenergic activation is involved in regulating the strength and precision of episodic-like memory. These findings are relevant to investigations in humans with respect to whether the emotional impact of an experience not only influences the strength of declarative (episodic) memory, but is also associated with changes in memory accuracy, fidelity, and susceptibility to incorporation of misinformation (Morgan et al., 2004; Porter et al., 2008; Smeets et al., 2009; Hoscheidt et al., 2014).

New neuroscience technologies increase the need to investigate the exact role of brain regions in memory. *In vivo* neuroimaging, optogenetics, and electrophysiological studies as well as molecular studies often show regional differences in brain activity after training that are difficult to interpret with general memory tasks. This is nicely illustrated by findings that drug administration after inhibitory avoidance training into a variety of brain regions, e.g., basolateral amygdala, hippocampus, dorsal striatum, insular cortex, anterior cingulate cortex and prefrontal cortex, induces highly comparable retention enhancement (Roozendaal and McGaugh, 2011). Thus, although these findings clearly show that these brain regions are all involved in regulating memory of inhibitory avoidance training, a possible specific role of these brain regions in particular aspects of information processing cannot be discerned. Experiments using context preexposure protocols have indicated that inhibitory avoidance can be dissociated into contextual and aversive components. Thus, *N*-Methyl-D-aspartic acid (NMDA) receptor blockade in the hippocampus after training does not impair inhibitory avoidance retention of rats preexposed to the training context, suggesting that hippocampal NMDA receptors are required for consolidation of a memory for the context, but not for context-shock association, and supporting the view that inhibitory avoidance is based upon an association between context and footshock (Roesler et al., 1998, 2003). The findings of the present report are consistent with this view, since they show that inhibitory avoidance is strongly dependent on context discrimination. In fact, subtle changes in the training apparatus resulted in a clear effect on discrimination at testing. Other studies have also used a modified inhibitory avoidance protocol to investigate the involvement of different brain regions in processing contextual and aversive components of inhibitory avoidance training. Inhibitory avoidance can be learned if rats are first exposed to the context and then, on a subsequent day, given a brief footshock in that context (Liang, 2001). Consistent with a role for the hippocampus in context memory, it was found that the non-selective muscarinic cholinergic agonist oxotremorine administered into the hippocampus after context exposure enhanced the subsequent conditioning whereas infusions administered after the footshock training were ineffective (Malin and McGaugh, 2006). In contrast, oxotremorine infused into the rostral anterior cingulate cortex selectively enhanced memory when administered after the footshock training. Oxotremorine infused into the basolateral amygdala enhanced retention when administered after either the context or footshock training, indicating a dissociable role of these brain regions in the processing of different aspects of inhibitory avoidance memory. We have shown in the present study that posttraining noradrenergic activation with yohimbine enhances memory of the association of footshock with the specific training context. Since the learning occurs within a single trial, the inhibitory avoidance discrimination task is suitable to study the strength and accuracy of episodic-like and contextual memory in combination with complementary molecular or physiological approaches in order to understand the contribution of different brain regions to different aspects of memory processing.

### Conflict of interest statement

The authors declare that the research was conducted in the absence of any commercial or financial relationships that could be construed as a potential conflict of interest.
